# Considerations about Hypoxic Changes in Neuraxis Tissue Injuries and Recovery

**DOI:** 10.3390/biomedicines10020481

**Published:** 2022-02-18

**Authors:** Simona Isabelle Stoica, Coralia Bleotu, Vlad Ciobanu, Anca Mirela Ionescu, Irina Albadi, Gelu Onose, Constantin Munteanu

**Affiliations:** 1 Faculty of Medicine, University of Medicine and Pharmacy “Carol Davila” (UMPCD), 020022 Bucharest, Romania; stoica.simona@umfcd.ro (S.I.S.); anca.ionescu@umfcd.ro (A.M.I.); 2 Teaching Emergency Hospital “Bagdasar-Arseni” (TEHBA), 041915 Bucharest, Romania; 3 Stefan S. Nicolau Institute of Virology, 030304 Bucharest, Romania; cbleotu@yahoo.com; 4 Computer Science Department, Politehnica University of Bucharest (PUB), 060042 Bucharest, Romania; vlad.ciobanu@cs.pub.ro; 5 Teaching Emergency County Hospital “Sf. Apostol Andrei”, 900591 Constanta, Romania; irina.albadi@yahoo.com; 6 Faculty of Medicine, “Ovidius” University of Constanta, 900470 Constanta, Romania; 7 Department of Research, Romanian Association of Balneology, 022251 Bucharest, Romania; 8 Faculty of Medical Bioengineering, University of Medicine and Pharmacy “Grigore T. Popa”, 700115 Iasi, Romania

**Keywords:** hypoxia, ischemia, neuraxis, hypoxic-ischemic injuries, neural ischemia, neural tissue hypoxic injuries, neuro-recovery, neurorehabilitation

## Abstract

Hypoxia represents the temporary or longer-term decrease or deprivation of oxygen in organs, tissues, and cells after oxygen supply drops or its excessive consumption. Hypoxia can be (para)-physiological—adaptive—or pathological. Thereby, the mechanisms of hypoxia have many implications, such as in adaptive processes of normal cells, but to the survival of neoplastic ones, too. Ischemia differs from hypoxia as it means a transient or permanent interruption or reduction of the blood supply in a given region or tissue and consequently a poor provision with oxygen and energetic substratum-inflammation and oxidative stress damages generating factors. Considering the implications of hypoxia on nerve tissue cells that go through different ischemic processes, in this paper, we will detail the molecular mechanisms by which such structures feel and adapt to hypoxia. We will present the hypoxic mechanisms and changes in the CNS. Also, we aimed to evaluate acute, subacute, and chronic central nervous hypoxic-ischemic changes, hoping to understand better and systematize some neuro-muscular recovery methods necessary to regain individual independence. To establish the link between CNS hypoxia, ischemic-lesional mechanisms, and neuro-motor and related recovery, we performed a systematic literature review following the” Preferred Reporting Items for Systematic Reviews and Meta-Analyses (PRISMA”) filtering method by interrogating five international medical renown databases, using, contextually, specific keywords combinations/”syntaxes”, with supplementation of the afferent documentation through an amount of freely discovered, also contributive, bibliographic resources. As a result, 45 papers were eligible according to the PRISMA-inspired selection approach, thus covering information on both: intimate/molecular path-physiological specific mechanisms and, respectively, consequent clinical conditions. Such a systematic process is meant to help us construct an article structure skeleton giving a primary objective input about the assembly of the literature background to be approached, summarised, and synthesized. The afferent contextual search (by keywords combination/syntaxes) we have fulfilled considerably reduced the number of obtained articles. We consider this systematic literature review is warranted as hypoxia’s mechanisms have opened new perspectives for understanding ischemic changes in the CNS neuraxis tissue/cells, starting at the intracellular level and continuing with experimental research to recover the consequent clinical-functional deficits better.

## 1. Introduction

Without oxygen, many species on Earth would not survive because molecular oxygen is indispensable for biochemical and bioenergetic cellular processes [1,2]. Therefore, hypoxia is defined as the decrease or deprivation of oxygen in organs, tissues, and cells by decreasing oxygen supply (due to damage to the vascular network— in case of ischemia; due to anemia or other lack of oxygen conditions) or increasing oxygen consumption (as in the sudden increase in the rate of cell proliferation) [3,4].

Hypoxia can be physiological (with beneficial effects on the nervous system, respiratory system, cardiovascular system, and different metabolisms) or pathological (neoplasms, rheumatoid arthritis, and atherosclerosis) [5,6]. Although the brain represents only 2% of the body’s weight, it is the organ that consumes the most energy, needing at least 20% of the total oxygen to function normally [7]. Furthermore, it seems that the medullary blood supply is similar to the cerebral one, with a blood flow of 5:1 between the white substance and the grey matter, and the mechanisms of cerebral and medullary vascular self-regulation are independent of blood pressure values. In contrast, systemic variations in blood gases alter the medullary vascular flow (without redistributing it) [8,9]. Therefore, reducing the amount of oxygen can be harmful to nerve tissue, leading to neurological disorders, with significant medical and socio-economic implications.

Each cell and tissue has characteristic abilities to adapt to hypoxia conditions by stabilizing HIF alpha and regulating the various genes involved in angiogenesis and oxygen transport [6].

In 2019, the Nobel Prize for Medicine and Physiology was awarded for clarifying the molecular mechanism of cell sensitivity to oxygen, with implications in cell physiology and the pathophysiology of complex processes such as metabolic adaptation, neovascularization, and tumor progression. The mechanisms of molecular biology through which hypoxia influences cellular activity are difficult to understand, their complete elucidation being performed only recently, when the correlations between cellular oxygenation level and hypoxia-inducible factor (HIF), HIF inhibiting factor (FIH), hypoxia regulating element (HRE), Von Hipple-Lindau protein (VHL), proline hydroxylase (PHD) were understood, along with all cellular elements modulated by these factors [10].

Perinatal hypoxic-ischemic lesions produce brain injuries. About 40% of newborns do not survive, and 30% develop permanent neurological disorders (cerebral palsy, visual disturbances, epilepsy, neuro-cognitive delay, learning disorders). Hypoxic-ischemic lesions from perinatal nerve distress cause energy disorders in cell metabolism, leading to cell death through apoptosis, necrosis, and autolysis (autophagic cell death) [11].

Experimentally, it has been observed how prenatal hypoxia affects the migration of embryonic neuroblasts with the subsequent impairment of the development of the CNS in rats [12,13]. Furthermore, experimental ischemia in mice, through MCAO (middle cerebral artery occlusion—model of stroke), is associated with immune cells presence in the meningeal vessels and, respectively, ”the mice with MCAO showed an invasion of LysM GFP+ cells into the brain parenchyma especially in the peri-infarct region” [14].

On the other hand, in human adults, vascular disorders (such as stroke) are risk factors for neurodegenerative diseases [15], like Parkinson’s disease [16], dementia (and other cognitive impairments). In addition, there are also innate metabolic disorders that affect the CNS’s functioning, such as hyperhomocysteinemia (a consequence of impaired homocysteine metabolism or other cofactors involved in its degradation), which is an independent risk factor for stroke [17,18] occurrence [19,20].

Considering the implications of hypoxia on nerve tissue cells that go through different ischemic processes, in this paper, we will detail the molecular mechanisms by which cells feel and adapt to hypoxia. We will present the hypoxic mechanisms and changes in central nervous tissue. We also aim to evaluate acute, subacute, and chronic central nervous hypoxic-ischemic changes in the hope of discovering or improving some methods of neuro-muscular recovery necessary to regain individual independence.

## 2. Method

The documentation afferent to this paper (about the relationship between the processes of ischemia—hypoxia—recovery in the central nervous system—CNS) relies on both works freely identified in the literature and a rigorous related selection within a systematic literature review, following the “PRISMA” paradigm. Thereby, we interrogated the following medical databases: Elsevier, NCBI/PubMed, NCBI/PMC, PEDro, and—to check whether the (initially) found articles are published in ISI indexed journals—ISI (Institute for Scientific Information—ex Thomson Reuters—currently administered by Clarivate Analytics).

Our search referred to the period 1 January 2016 to 31 December 2020. We considered only works published in English and issued in ISI-indexed journals. Accordingly, we used—contextually—a series of key word combinations/”syntaxes”: ” Hypoxia” + ”nerve tissue” + ”lesion(s)”, ”Hypoxia” + ”nerve tissue” + ”injury(es)”, ”Hypoxia” + ”nerve tissue” + ”recovery”, ”Hypoxia” + ”nerve tissue” + ”cellular mechanisms”, ”Hypoxia” + ”CNS” + ”lesion(s)”, ”Hypoxia” + ”CNS” + ”lesion(s)”, ”Hypoxia” + ”CNS” + ”injuries(es)”, ”Hypoxia” + ”CNS” + ”injury(es)”, ”Ischemia” + ”nerve tissue” + ”lesion(s)”, ”Ischemia” + ”nerve tissue” + ”injury(s)”, ”Ischemia” + ”nerve tissue” + ”recovery”, ”ischemia” + ”nerve tissue” + ”cellular mechanisms”, ”Ischemia” + ”CNS” + ”lesion(s)”, ”Ischemia” + ”CNS” + ”lesion(s)”, ”Ischemia” + ”CNS” + ”injuries(es)”, ”Ischemia” + ”CNS” + ”injury(es)” The articles thus found have then been filtered in five steps (without meta-analysis), on the standardized base of the PRISMA inspired selection methodology.

## 3. Results

**Figure 1:**
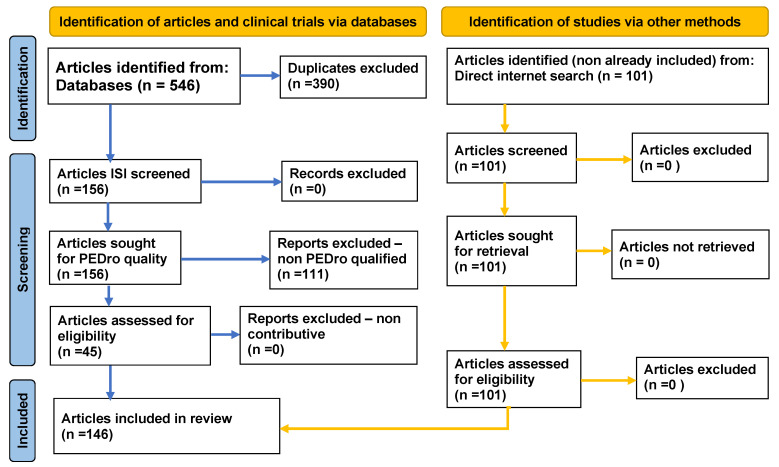
PRISMA flow diagram adapted to our study.

The PRISMA standardized methodology for achieving systematic reviews requires specific steps and a high level of strictness, which we have respected, resulting in 45 eligible and contributive works (see Figure 1 and Table S1). Although our systematic literature review has been thoroughly conducted, some bibliographic resources of interest still might have been overlooked. However, considering the contribution mentioned above of the freely discovered publications, hopefully, we have adequately covered the necessary information, as presented in the skeleton structure (see Table 1 at the end).

### 3.1. The Intimate Mechanisms of Hypoxia

**Figure 2:**
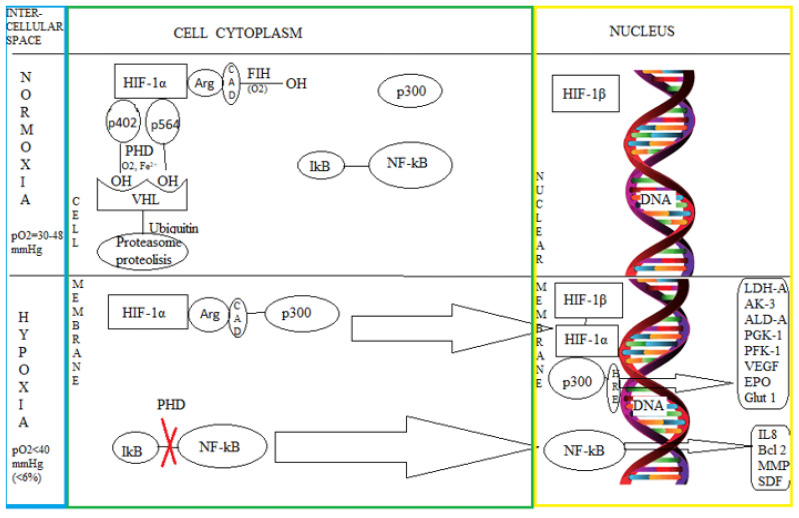
The cellular mechanism of hypoxia (showing how oxygen partial pressure, pO2, influences deoxynucleic acid, DNA, via hypoxia-inducible factor, HIF; with involvement of factor inhibiting HIF, FIH; arginine, ARG; C-terminal transactivation domain, CAD; protein 300, p300 and Von Hipple-Lindau factor, VHL and proline hydroxylase, PHD. We can see how hypoxia response elements (HRE) are activated, as the atypical Nuclear Factor kB, NF-kB, activation pathway, with involvement of inhibitor kB, I-kb. This triggers the genes of lactate dehydrogenase A, LDH-A; adenylate kinase 3, AK-3; aldolase A, ALD-A; phosphoglycerate kinase 1, PGK-1; phosphofructokinase, liver type, PFK-L 6; vascular endothelial growth factor, VEGF; erythropoietin, EPO; glucose transporter 1, Glut-1; interleukin 8, IL 8; B cell lymphoma-2, Bcl-2; matrix metalloproteases, MMP; stromal cell-derived factor 1, SDF).

HIF plays a central role in detecting and adapting cells to oxygen by transcriptionally activating genes controlling oxygen homeostasis and metabolic activation (Figure 2) [21]. There are several cytoplasmic HIF isoforms: HIF-1α and HIF-2α (activating the HRE’s transcription, without redundant activity) and HIF-3α (the most distant isoform which, in some cases, may encode a polypeptide that inhibits the expression of HRE-dependent genes). HIF-2α is present in tumor cells (such as those in clear-cell renal cell carcinoma associated with von Hippel-Lindau disease), and HIF-1α has been found in normal cells of the human body. However, it appears that activation of the erythropoietin production gene (EPO) is preferentially achieved under the action of HIF-2α (also occurring through activation produced by HIF-1α) [3,22].

HIF-1 is essential for normal development and the response to ischemia/hypoxia, tumor development, energy metabolism, angiogenesis, apoptosis, proliferation, and vasomotor function [23]. HIF 1 binds many genes that contain in their structure hypoxic response elements such as vascular endothelial growth factor (VEGF), glucose transporter 1 (GLUT-1), adenylate kinase 3 (AK-3), aldolase A (ALD-A), phosphoglycerate kinase 1 (PGK-1), 6-phosphofructokinase, liver type (PFK-L), and lactate dehydrogenase A (LDH-A) [24].

The HIF-DNA complex is a heteromer, the binding being achieved by the subunits α and β. While the expression of the HIF-1β subunit is constitutive, the presence of the HIF-1α-subunit increases exponentially with the decreases of cellular oxygenation below 6%. That means an oxygen partial pressure of 40 mmHg measured at sea level when the average partial pressure of the oxygen in the nervous tissue is 30–48 mmHg [25,26,27]. The amino-terminal end of HIF-1α is sufficient for dimerization with the HIF-1β subunit and DNA binding [25]. Cellular sensitivity to oxygen is ensured by the activity of the enzyme proline hydroxylase (having 3 isoforms), which, in the presence of iron ions and oxygen, adds two hydroxyl groups to the HIF-1α subunit (to 2 terminal proline residues: Pro-402 and Pro-564) [10,28,29]. At the same time, the oxygen-dependent hydroxylation, performed by FIH to arginine residue of HIF-1α C-terminal transactivation domain (CAD), cancels the HIF-1α interaction with p300, preventing its translational activity [27,30].

Under normal oxygenation, the HIF-1α protein subunit is rapidly degraded by proteosomes. The whole process is mediated by the Von Hipple-Lindau protein factor (pVHL), an E3 ubiquitin recognition and binding component that promotes ubiquitin-dependent proteolysis of this subunit [31]. Under hypoxic conditions, the degradation of the HIF-1α subunit is suppressed, leading to gene transcription activation. At the experimental level, cobalt ions and iron chelators simulate the conditions of cellular hypoxia. In their presence, the binding of HIF-1α to pVHL is prevented [10,32].

HIF-1 is also found in mammalian cell cultures raised under low oxygen pressure conditions and is needed to amplify the transcription of genes that mediate erythropoietin (EPO) production in hypoxic cells [33,34,35]. Oxygen is essential for mammalian life through oxidative phosphorylation and adenosine triphosphate (ATP) synthesis [36,37].

In response to hypoxia, HIF-1 mediates the activation of anaerobic glycolysis pathways, erythropoietin synthesis pathways (in anemia or individuals living at altitude), and production of VEGF (with new blood vessel formation as in chronic myocardial ischemia). HIF influences genes such as VEGF, EPO by binding to the HRE gene sequence (thus, HRE shows HIF activity) [6]. Molecular mechanisms that mediate the cellular response to hypoxia have been studied regarding erythropoietin production, which controls erythrocytes formation (and increased tissue oxygenation) through specific growth factors. Hypoxia conditions (growth of cell cultures in the atmosphere with 0.1% oxygen, in the presence of cobalt chloride or deferoxamine) determine the binding to the cis end of the EPO3 gene of the trans region of the α subunit of HIF-1. In conditions of deficient oxygenation obtained by treating the cell cultures with deferoxamine or cobalt chloride, the EPO3 gene expression has cell specificity based on HIF-1α-mediated activation [38]. The persistence of HIF-1α in cells grown in hypoxemic environments is due to the binding of cobalt ions and deferoxamine to CAD located at the carboxy-terminal end of HIF-1 [25].

**Figure 3:**
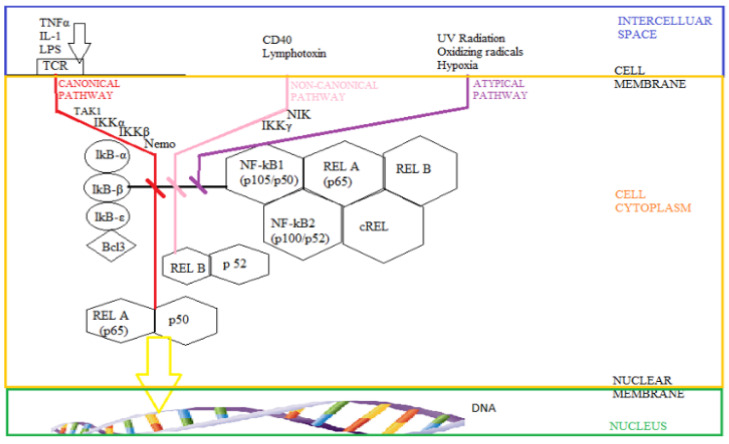
The Nuclear Factor kB (NF-kB) pathways activation: the canonical pathway (triggered by the action of tumor necrosis α, TNFα; interleukin 1, IL-1, and lipopolysaccharides, LPS, on T cell receptor, TCR, with involvement of transforming growth factor activating kinase—B, TAK1; the kB inhibitor complex kinase α, β, IKK α, β; of the essential modulator of NF-kB, IkB-α, β, ε kB inhibitor family, Nemo), the non-canonical pathway (involving NF-κB-induced kinase, NIK, and IKK γ) and the atypical pathway.

Nuclear Factor kB (NF-kB) is a major transcription factor under stress, being activated (see Figure 3) by hypoxia or decreased oxygen availability [39]. NF-kB is part of a family of transcription factors composed of RelA, RelB, cRel, NF-kB1 (p105/p50), and NF-kB2 (p100/p52) [40]. These transcription factors are maintained in the cytoplasm in an inactive form by the kB inhibitor family’s action (IkB) [41]. The stimulation produced by various stressors leads to the accumulation of NF-kB in the nucleus and its binding to DNA, using some paths classified as canonical, non-canonical, and atypical. The most studied pathway is the canonical (or classical) activation of NF-kB. This one involves the activation of transforming growth factor activating kinase-B (TAK1) and the kB inhibitor complex kinase (IKK), composed of IKKα, IKKβ, and IKKγ or the essential modulator of NF-kB (Nemo) [41]. The non-canonical NF-κB activation pathway consists of NF-κB-induced kinase (NIK) and the IKKγ activating homodimer [42,43]. The atypical NF-kB activation pathway usually does not require the presence of the IKK complex and acts directly on the IkB [41]. NF-κB activation pathways are strongly influenced by changes in ubiquitin components [39].

A ligand for the receptor, such as cytokines or foreign deoxyribonucleic acid (DNA) and ribonucleic acid (RNA), is usually required to activate most NF-κB pathways. Stimulation by damaged DNA activates sensitive NF-kB kinase in the nucleus, followed by cytoplasmic growth of IKK. When activating HIF, the cell uses oxygen sensors such as PHD and FIH. PHD links oxygen sensitivity to NF-kB activation, acting directly on IKK. At the same time, PHD1 was identified as the enzymatic isoform with the highest level of control of IKK activity [39]. Most studies have investigated the activation of NF-κB in response to hypoxia in neoplastic cells and have shown a decrease in apoptosis and increased angiogenesis.

However, the situation is different in tissues such as the brain and myocardium, in which NF-kB’s role appears to be more complex through the induction and suppression of apoptosis depending on the context [39]. Under hypoxic conditions, NF-kB modulates the expression of numerous proteins involved in controlling apoptosis (such as members of the Bcl-2 family) and the inhibition of programmed cell death [44,45,46]. In addition, NF-kB increases the expression of IL-8, an essential cytokine in inducing angiogenesis that contributes to the generation of neovascularization in hypoxia [47,48]. NF-kB also induces the expression of proteins involved in motility and adhesion, such as matrix metalloproteases (MMP) and stromal cell-derived factor 1 (SDF) in tumor, immune and neuronal cells [46,49]. Therefore, the hypoxia-induced NF-kB factor is essential for many cellular responses to this stimulus, (especially) prevention of apoptosis, induction of angiogenesis, and promotion of cell motility [39,50,51].

### 3.2. The Influence of Hypoxia on the Nervous Tissue

Hypoxia complexly influences the physiology of all types of cells, including the nerve cells, in all stages of nerve tissue development (starting with the neonatal period and ending with that of old age), as well as in various traumatic conditions (spinal cord injury, traumatic brain injury) and non-traumatic conditions (stroke, chronic pain, epilepsy, certain congenital or acquired neurodegenerative diseases). Hypoxia has some pathological consequences on the CNS (CNS) in different post-lesional stages divided into acute (in the first 2 weeks), subacute (in the next 3–11 weeks), early chronic (for the 12–24 next weeks), and chronic (for a more extended period than 24 weeks) [52,53,54].

Nerve tissue comprises neurons and glial cells, such as microglia or astrocytes [55]. Microglia are derived from erythromyeloid progenitors migrated to the brain during intrauterine life [56]. Microglia have an essential role in defending against microbial aggression and CNS aggression and synaptic budding, neurogenesis, and cerebral homeostasis [57,58]. It has also been observed that, among flavonoids, wogonin and baicalein inhibit microglial inflammatory activity (by decreasing the production of nitric oxide (NO) and the inducible activity of NO synthase and NF-κB activation) [59]. In addition, melatonin is a pineal hormone with an anti-inflammatory effect after stroke (by clearing ROS and inhibiting the inflammatory response) in microglia [60].

Astrocytes are essential in maintaining the integrity of the blood-brain barrier and the perineuronal environment; in the metabolism of glutamate (and decreased excitotoxicity), in the preservation of calcium and potassium homeostasis in the extracellular environment [61]. In addition, astrocytes have a critical homeostatic role in the CNS through neurogenesis, neuroprotection, immunomodulation, and their antioxidant role [62]. It has also been found that HIF1α stimulates the production of peroxisomes (organelles with essential functions in glial cell metabolism) [62].

In the glial cell membranes, Aquaporin 1 (AQP-1) water channel [63] is very little expressed in white and grey matter (in astrocytes ependymal cells and thin fibers in the posterior medullary horns of the normal CNS), act as an ion channel in the secretion of cerebrospinal fluid and is well expressed in choroid plexus cells [64].

At the nuclear level, the High mobility group box 1 (HMGB1) protein is present in all cells, having a role in stabilizing and repairing deoxyribonucleic acid (DNA) [65]. HMGB1 is released from necrotic neurons or is actively secreted from microglia, monocytes/macrophages, and neutrophils, mediating the neuroinflammatory response and contributing to the pathogenesis of ischemic stroke [66]. Extracellular HMGB1 binds to different membrane receptors as toll-like receptors (TLR-2, TRL-4). HMGB1 can be altered by redox reactions with implications for acute and chronic cellular changes in stroke [67,68].

The mechanisms of hypoxia act in a very complex manner on the CNS through all the molecules it involves. In this way, erythropoietin has a neuroprotective effect, engaging in antiapoptotic, anti-inflammatory, neuro-neurovascular remodeling, and stem cell proliferation mechanisms [69].

At the mitochondrial level, hypoxia causes the reprogramming of the functioning of the respiratory chain by changing (transient and reversible) the path of nicotinamide adenine dinucleotides oxidation (NAD) of the substrate (complex I) to the oxidation of succinate (complex II). Activation of the respiratory chain complex II is a vital hypoxia adaptation mechanism required for the production of cellular energy by succinate (for immediate resistance of the body), stabilization of HIF-1α by succinate and initiation of its transcriptional activity for long-term adaptation, and activation of the GPR91 succinate receptor (G protein receptor 91) [70]. This mechanism of activation of the respiratory chain complex II has the following regulatory functions: sensitization and adaptation to the gradual decrease of oxygen in the environment (for the selection of the most efficient ways of oxidation of the substrate in hypoxia), compensation (with the immediate formation of the body’s response and resistance to hypoxia), transcriptional (with activation of HIF-1α synthesis and genes that ensure adaptation to hypoxia for long periods), receptor (for mitochondrial participation in the GPR91 succinate receptor-mediated intercellular signaling system) [71,72,73].

A specific neural pathway for adapting to hypoxia has been discovered, which determines the increase in the biosynthesis of cellular fatty acids with their subsequent esterification. Experimentally, it was observed that the inhibitors of fatty acid synthesis (such as Acetyl-Co-A carboxylase and fatty acid synthetase) increased the ratios of NADH^+2^/NAD^+^ and NADPH^+2^/NADP^+^ in hypoxic conditions. Inhibition of fatty acid synthetase increased lactic acid levels in normoxia and hypoxia. They show that fatty acids can be proton acceptors from anaerobic glycolysis under hypoxic conditions [74].

### 3.3. Newborn Hypoxic-Ischemic Encephalopathy

Perinatal hypoxic ischemia is the leading cause of cerebral distress in newborns. Therefore, this condition can have significant individual and social implications [75,76].

Hypoxic-ischemic brain lesions in the newborn are significantly influenced by inflammation, activating neural cells, and local infiltration of circulating leukocytes. An initial inflammatory response is followed by a secondary one (which can last for several days) and a subsequent anti-inflammatory reaction. The pathophysiology of hypoxic-ischemic encephalopathy is based on reduced blood flow and brain oxygenation. Mitochondria tend to hyperpermeability in response to the process of hypoxia-ischemia, and this excessive permeabilization of the mitochondrial membrane is considered a point of irreversibility in the apoptosis following perinatal hypoxic encephalopathy [11,77,78].

Severe hypoxic-ischemic lesions initially cause energy deficiency followed by primary neuronal death, correlated with the decrease in ATP and the increase in lactic acid production intracellular level associated with an increase in the level of reactive oxygen species (ROS). ROS are involved in cell physiological processes but can cause cell damage, inflammation, and oxidative stress [79]. Thus, rapid swelling and cell necrosis occur. Severe hypoxic-ischemic lesions shorten the period of revascularization and cerebral metabolic recovery, leading to secondary lesions (at least 6 h after the initial lesion) with delayed neuronal apoptosis (related to excitotoxicity, oxidative stress, and inflammation) [11,80].

Hypoxic-ischemic lesions of the newborn’s brain intensely activate microglial cells, which are involved in secondary energy disorders through the production of proinflammatory cytokines (TNF-α, IL-1b, IL-6, and IL-18), complement system factors, excitotoxic amino acids. On the other hand, microglia are involved in the relief of inflammation and repair processes after suffering from hypoxic-ischemic encephalopathy by phagocytosis processes of cell debris [81,82].

Proinflammatory cytokines and ROS resulting from damage of hypoxic-ischemic neurons [11] may activate reactive astrogliosis and delay local production of proinflammatory cytokines (TNF-α, IL-1a, and b IL-6). It appears that reactive astrogliosis also produces anti-inflammatory cytokines (IL-9, IL-10, and IL-11), promoting tissue healing by activating Toll-like receptor 3 (TLR3). As a result of neonatal hypoxic-ischemic brain damage, T lymphocytes can also enter the CNS releasing micro RNA 210 (miR-210) due to transient focal ischemia [83]. MiR-210 inhibits HIF-1α by performing a negative feedback loop in hypoxic differentiation of T lymphocytes [84]. In other words, acute or chronic suffering in the CNS causes the release of adenosine triphosphate (ATP), which in the intercellular space has an immediate excitotoxic effect by coupling with astrocyte receptors such as P2X7 (ion gate type) and P2Y1 (G protein-coupled receptor) following astrogliosis (which isolates the injured areas) and the synthesis of neurotrophic substances (necessary for neuronal recovery). It seems that purine mechanisms mediate astrogliosis following neurotrauma and local hypoxia/ischemia. In the long run, the effect of nucleotides is to amplify primary lesions, with involvement in chronic pain, epilepsy, and post-traumatic cell death [85].

### 3.4. Adult Brain Ischemic Vascular Lesions

Stroke (which can have multiple etiologies and is at higher risk of production in diabetic patients or after SARS COV2 infection) is a neurological condition that is becoming more common, with many individual, family, and social implications [86,87,88,89].

In adulthood, ischemic vascular lesions in stroke are risk factors for neurodegenerative diseases, and systemic hypoxic episodes increase the production and accumulation of Aβ proteins along with the decreased expression of neprilysin (NEP) [90]. The decrease of NEP, the primary enzyme that degrades Aβ proteins, affects the clearance of this protein. On the other hand, Aβ proteins derived from the transmembrane domain of amyloid precursor protein (APP), together with other active metabolites (including the C-terminal fragment in the intracellular domain of APP), regulate the expression of NEP and other neuronal genes. Some studies have also shown that caspase activation may be necessary for regulating brain NEP in hypoxic and ischemic conditions, and the decrease of the Aβ proteins elimination increases the risk of Alzheimer’s dementia [15,91,92]. In the acute phase, post-stroke VEGF increases the permeability of the brain-blood barrier; in the chronic phase, this molecule promotes neurogenesis and cerebral angiogenesis [93]. In patients with hyperhomocysteinemia (a genetic disorder involving cellular hypoxia), neural cells are sensitive to prolonged exposure to the elevated level of homocysteine, due to the adverse effects of the reactive oxygen species and posttranslational changes in proteins (which occur later), and the adaptive CNS (survival) response to sublethal ischemia is also preserved [19].

Primary and secondary lesions occur in traumatic brain injury (TBI). Primary lesions are triggered by endogenous changes such as oxidative stress, glutamate-related excitotoxicity, immune response, disorders of ionic homeostasis, and increased vascular permeability, causing degeneration and neuronal apoptosis [94]. Secondary TBI lesions result from oxidative stress, by excessive accumulation of ROS that causes damage to cellular components (lipids, proteins, DNA), followed by impaired functioning and apoptosis of neuronal cells [95]. For its role in proliferation, signal transduction, and regulation, miRNA can also be considered a TBI biomarker [57,58,83,96,97,98].

### 3.5. Adult Spinal Cord Injury and Hypoxia

The dimensions of the modern world are moving at increasing speeds, and the risk of polytrauma (including TBI and SCI) is growing proportionately, with consequences that often are difficult to manage [87,99,100].

After SCI (similar to TBI), primary and secondary lesions appear. The initial injuries are consecutive to the direct traumatic impact (and are accompanied by neuro-vascular lesions), and secondary lesions subsequently occur through vascular dysfunction, inflammation, demyelination, and neuronal morphophysiological impairment [86,101].

In spinal cord injury (SCI) and TBI, the neural stem cells (NSCs) migrate and differentiate around damaged nerve tissue areas. In the case of SCI, the interstitial environment changes its composition due to transient hypoxia, accumulation of potassium ions, calcium, reactive oxygen species, and increase of glutamate activity. All these biochemical changes are not conducive to the survival of locally migrated NSCs. The granulocyte-macrophage colony-stimulating factor (GM-CSF), a cytokine that stimulates the differentiation and proliferation of hematopoietic cells, influences the nervous system’s functioning. After SCI, GM-CSF accumulates locally and ensures the survival of dopaminergic neurons, inhibits the formation of glial scars, having a neuroprotective role. Experimentally, GM-CSF may promote in vitro NSC proliferation and in vivo motor recovery in adult mice. Overexpression of the GM-CSF gene protects the NSC by increasing the resistance of these cells to apoptosis induced by hydrogen peroxide in hypoxia, thus ensuring the survival and differentiation of NSC in experimental SCI models [57,102,103,104].

It has been observed that VHL protein is increased at the medullary level after SCI, reaching a peak level at 3 days post-trauma. The increase of VHL protein was associated with neuronal apoptosis because it was not found in astrocytes and microglia. Decreased VHL levels may reduce glutamate-dependent neuronal apoptosis, but increased expression of VHL protein above a critical limit does not appear to produce further changes in neuronal apoptosis [31].

HIF-1α and VEGF expression in spinal cord injury areas after SCI was associated with the process of angiogenesis and improvement of local microcirculation. The main function of VGEF in the CNS is angiogenesis, which can protect nerve cells against ischemic and mechanical damage to the axons [105,106,107].

Increasing HIF-1α expression has a protective role in promoting functional recovery after spinal cord injury. Using DMOG-dimethyloxalylglycine treatment, it was obtained a sustained activation of HIF-1α by inhibition of prolyl hydroxylase. DMGO treatment significantly increases HIF-1α expression, inducing molecule stability that decreases apoptotic protein expression and promotes neuronal survival. This treatment also stimulates axonal regeneration by controlling the stability of microtubules in both in vivo and in vitro. On the other hand, DMGO promotes neuronal survival and axonal regeneration by activating the autophagy induced by the HIF-1α/BNIP3 signaling pathway. These experiments sustain the idea that the DMGO molecule can help treat patients with SCI [108]. It seems that amplification of autophagy reduces initial cell death by restricting the function of autophagy-associated genes and modulating the expression of inflammatory cytokines (TNF α, IL 1β) [109].

### 3.6. Hypoxia and Functional Recovery

Experiments with intermittent exposure to hypoxia have also been performed in relatively short sessions in patients with incomplete deficits after SCI. Respiratory, psychological, and motor function benefits were observed. In addition, following intermittent exposure to hypoxia of rats with SCI, a serotonin-dependent increase of brain-derived neurotrophic factor (BDNF) synthesis was observed in the areas of the anterior medullary horns containing the phrenic nerve nucleus; this could explain the improvement in respiratory function [110,111,112,113].

Other potential mechanisms of the beneficial exposure to intermittent hypoxia in SCI could be the increase of cytoglobin, the induction of heat shock protein 70 (HSP70), together with the rise of HIF-1α [114]. Hypoxic exposure (60 cycles of 30 s of intermittent hypoxia with 1.5% atmospheric oxygen, followed by 5 min of normoxia) induced HIF-1α accumulation, following the generation of reactive oxygen species by nicotinamide adenine dinucleotide phosphate (NADPH) oxidase. Also, in rodents exposed to intermittent hypoxia, there was increased gene expression of VEGF and increased growth hormone release [115,116].

This conditioning of neural activity by intermittent hypoxic stimulation is thought to stimulate neuroplasticity of the CNS [117,118,119]. It has also been found that sleep apnea syndrome (in its mild to moderate forms) is associated with better outcomes rather than intermittent exposure to hypoxia (in patients with incomplete SCI) [120].

It has also been observed that HIF-1α expression is increased during hypoxia or in the ischemia/reperfusion change after SCI, which could suppress the autophagy of neuronal cells. On the other hand, HIF-1α activation produces an anti-inflammatory effect (by decreasing TNF-α, IL-1β, IL-6, and IL-18 in SCI models in rats). In addition, intermittent hypoxia may induce HIF-1α expression, resulting in intermittent activation of autophagy (HIF-1α-dependent during the process of intermittent hypoxia) [121]. HIF-1α can cause additional expression of transcriptional factor p62, which can subsequently release from its binding to the Bcl-2 protein, activating it to participate in the autophagy process [38]. HIF-1α may be decreased in the first 24 h after SCI, just as it can be slightly lowered under normoxic conditions. It was also reported that HIF-1α had a significant increase after the first 24 consecutive hours of an SCI [38].

Erythropoietin is essential for the recovery of cognitive and memory disorders (following ischemic hypoxia) by inducing long-term synaptogenesis and repairing lesions of nerve terminals based on increased expression of synaptic proteins (synapsin 1 and postsynaptic density protein 9, PSD95) of the dendritic marker microtubule-associated protein 2 (MAP-2), of the axonal density and the decrease of a factor associated with the axonal injury, amyloid precursor protein (APP) [122].

Knowing the importance of mild chronic hypoxia in functional recovery by stimulating vascular remodeling in the brain, its implication in spinal vascular remodeling has been investigated after exposure to chronic mild hypoxia (8% O2, for 7 days) [123]. Thus, it was observed how chronic mild hypoxia promotes endothelial proliferation and increased vascularization by increasing angiogenesis and arteriogenesis markers such as rising vascular expression of fibronectin in the extracellular protein matrix, simultaneously with increased endothelial expression of the α5β1 integrin receptor of fibronectin and increased endothelial expression of the junctional proteins claudin-5, ZO-1 and occluding and astrocyte activation (Halder SK, 2018). It was noted that these changes in exposure to mild chronic hypoxia were more critical in the medullary white matter. Spinal blood vessels appear to have considerable remodeling potential, with α5β1 integrin essential in promoting endothelial proliferation [19,123].

Research has also been done on the effect of deferoxamine administration in the first 1–2 weeks after SCI in rats. Significant neovascularization was observed in the spinal cord injury [124], demonstrating increased expression of HIF-1α and VEGF. In addition, after deferoxamine treatment, rats with SCI showed a significant improvement in motor deficit, spared nerve tissue area, and electrophysiological conduction. However, all these favorable effects produced after deferoxamine treatment in post-SCI advance were suppressed by treatment with lenvatinib, a VEGF receptor inhibitor, suggesting that deferoxamine’s main pharmacological effect in SCI is to promote neovascularization by HIF-1α and VEGF overexpression [125].

It was also found that VEGF production is stimulated by neuropeptide Y (NPY), whose serum levels increase during exercise, hypoxia, cold exposure, tissue injury, ischemia, and hemorrhagic shock [126]. In addition, NPY is an orexigenic hormone whose insulin negatively regulates hypothalamic activity [127,128].

Erythropoietin appears to promote functional recovery after spinal cord injury (SCI). Studies regarding EPO efficiency have been performed both in vitro, on neural stem cells harvested from animals, and in vivo, on rats that have undergone spinal cord contusive patterns. In vivo results showed superior β-tubulin production in erythropoietin-treated neuronal and glial cells. Also, only rats with SCI treated with erythropoietin resumed gait compared to the control group [129]. The positive effects of erythropoietin are also found in recovery after traumatic brain injuries (TBI) [130].

The role of aquaporins in pathogenesis and post-SCI recovery was also studied. In SCI, AQP-1 is 4–8 times better expressed in the traumatic area, probably having a role in the appearance of post-traumatic edema and the formation of spinal cysts. Moreover, maintaining elevated AQP-1 values in the subacute and chronic post-SCI phases, similar to high HIF-1α values, may result in consecutive SCI hypoxia.

Another study highlighted the post-SCI protective role of glutamine synthetase, which metabolizes glutamate to glutamine. Thus, protection against hypoxia-induced excitability has been observed (with inhibition of decreased compound action potentials), probably by blocking gamma-aminobutyric acid A (GABA A) receptors [131].

An interesting fact also refers to the Mediterranean diet based on an abundant consumption of olive oil. Olive oil has anti-inflammatory and immunomodulatory effects on the nervous system via (poly)-phenols, which modulate the activity of NF-κB, HIF-1α, signal transducer, and transcriptional activator 3 (STAT3), and mitogen-activated protein kinases (MAPKs) [132,133].

Natural flavonoids (such as wogonin, baicalein, curcumin, apigenin, quercetin, luteolin) have an anti-inflammatory effect because they have been shown to inhibit the production of IL-6, TNF-α, and IL-1β from the MAPK pathway of the nervous system [59,134,135]. It should also be mentioned that a beneficial effect of cannabinoid receptor agonists on oligodendrocytes (and their precursor cells) has been studied, like apamin (bee venom) [136,137,138,139,140].

**Table 1:** PRISMA resulting conceptual skeleton structure of the article’s organization approach.

***The cellular mechanism of hypoxia***		
***Article***	***Ref. no***	***Subject***
(Thornton, 2017)	[11]	Hypoxic-ischemic lesions cause energy disorders in cell metabolism, leading to cell death through apoptosis, necrosis and autolysis
(Cai, 2019)	[14]	MCAO mice showed an invasion of immune cells into the brain
(Nowak-Sliwinskaet, 2018)	[22]	HIF-1 is essential for normal development and the response to ischemia/hypoxia, tumor development, energy metabolism, angiogenesis, apoptosis, proliferation, and vasomotor function
(Yuniati, 2019)	[43]	NF-kB modulates the expression of numerous proteins
(Gschwandtner, 2019)	[44]	apoptosis and the inhibition of programmed cell death
(Yang, 2017)	[47]	NF-kB increases the expression of IL-8, inducing angiogenesis that contributes to the generation of neovascularization in hypoxia
2. ***The influence of hypoxia on the nervous tissue***		
(Clark, 2019)	[54]	Nerve tissue is made up of neurons and glial cells
(Miller, 2017)	[55]	Microglia are derived from erythromyeloid progenitors
(Greenhalgh, 2018)	[56]	Microglia have an essential role
(Barrett, 2017)	[57]	cerebral homeostasis
(Ginwala, 2019)	[58]	NO synthase and NF-κB activation
(Liu, 2019)	[59]	Melatonin is a pineal hormone with anti-inflammatory effect
(Becerra-Calixto, 2017)	[60]	calcium and potassium homeostasis
(Islinger, 2018)	[61]	HIF1α stimulates the production of peroxisomes
(Gorgulho, 2019)	[63]	High mobility group box 1 (HMGB1) protein
(Kim, 2017)	[64]	neuroinflammatory response, pathogenesis of ischemic stroke
3. ***Newborn hypoxic-ischemic encephalopathy***		
(Geisler, 2019)	[75]	reduced blood flow and brain oxygenation
(Rohowetz, 2018)	[76]	Mitochondria tend to hyperpermeabilize
(Weiskirchen, 2016)	[77]	ROS are involved in cell physiological / pathological processes,
(de Faria, 2019)	[80]	phagocytosis processes of cell debris
4. ***Adult brain ischemic vascular lesions***		
(Carvajal, 2016)	[85]	The ionotropic glutamate receptor AMPA
(Galicia-Garcia, 2020)	[86]	Stroke (neurological condition) - individuals, family and social
(Pennisi, 2020)	[87]	Stroke neurological condition and SARS-CoV-2
(Shahabipour, 2017)	[88]	Aβ proteins along with the decreased expression of neprilysin
(Tanaka, 2020)	[90]	Aβ proteins - Alzheimer's dementia
(Şekerdağ, 2018)	[91]	acute phase post stroke VEGF increases permeability of BBB
(Morya, 2019)	[92]	Primary and secondary lesions occur in traumatic brain injury
(Ramirez, 2018)	[94]	proliferation, signal transduction
(Iraci, 2016)	[95]	regulation, miRNA - traumatic brain injury
(Ciregia, 2017)	[96]	Traumatic brain injury (TBI) biomarker
5. ***Adult spinal cord injury and hypoxia***		
(Poniatowski, 2017)	[98]	risk of polytrauma
(Lin, 2020)	[99]	primary and secondary lesions
(Kim, 2019)	[102]	Overexpression of the GM-CSF gene protects
6. ***Hypoxia and functional recovery***		
(Miranda, 2019)	[111]	intermittent exposure to hypoxia
(Zhou, 2016)	[117]	hypoxic stimulation is thought to stimulate neuroplasticity
(Ke, 2019)	[119]	intermittent hypoxia may induce HIF-1α expression
(Tan, 2018)	[124]	VEGF production is stimulated by neuropeptide Y (NPY)
(Yung, 2020)	[126]	NPY is an orexigenic hormone, negatively regulated by insulin
(Gaforio, 2019)	[130]	Mediterranean diet based on an abundant consumption of olive oil
(Angeloni, 2017)	[131]	olive oil has anti-inflammatory and immunomodulatory effects
(Libro, 2016)	[132]	Natural flavonoids (wogonin, curcumin, apigenin, quercetin)
(Teleanu, 2019)	[133]	anti-inflammatory effect
(Ilyasov, 2018)	[134]	inhibit the production of IL-6, TNF-α, and IL-1β - MAPK pathway
(Gu, 2020)	[137]	apamin (bee venom)
(Cramer, 2020)	[138]	cannabinoid receptor agonists on oligodendrocytes

## 4. Discussion and Conclusions

In the last decade, huge scientific research efforts have been deployed to acquire a better understanding of CNS lesions and to significantly improve the clinical-function outcomes, including management, because of their dreadful, multiplane life-threatening, and disabling potential, but at least for now, there are still no medical (of any kind) interventions [17,18,141] able to cure or decisively contribute to their healing [14,140].

Under these circumstances, enhancing the thorough approach of this very complex and challenging pathology domain must be continued without omitting any possible contribution. Serving this purpose, for instance, there are to be found in the literature the beneficial effects in restoring the nervous system through the action of natural plant substances on the mechanisms of hypoxia [140].

It is known that hypoxia induces various adaptive and survival changes for both normal and tumor cells. However, there are essential differences between hypoxic mechanisms in normal cells compared to tumor ones. Therefore, further hypoxia is critical for recovery from ischemic disorders in neuraxis tissue, for instance, by modulating the related involvement of BDNF [141,142,143], cytoglobin [114], HSP70, VEGF, erythropoietin, fibronectin.

In contrast, in tumoral tissue, due to the uncontrolled cell proliferation and relative low vascularization—ischemia—it results in cell oxygen-starvation—hypoxia. Even if hypoxia inhibits normal cell development, neoplastic cells develop, and hypoxic mechanisms are used for tumor proliferation (although unsystematized tumor development can cause central necrosis and cell death) [144]. All neoplastic ischemic changes, called “pseudo-hypoxia”, are essential for angiogenesis, growth, and tumor metabolic adaptation, including thorough the NF-kB way and resistance to treatment. The discovery of links between hypoxia and neoplastic metabolism has led to the development of immuno-oncology (and the synthesis of cytostatics that specifically inhibit specific pathways of pseudo-hypoxia like VEGF inhibitors HIF-2α inhibitors) [145,146].

As mentioned above, the functional recovery of CNS lesions appears to be positively influenced by the activation of normal hypoxia pathways.

Considering all the above, the study regarding hypoxia’s mechanisms and ischemia mechanisms targeted to emphasize and synthesize the actual perspectives in understanding ischemic related CNS changes, especially at the intimate level involved in lesions development and or (dialectical, paradoxically) recovery.

Thus, it was observed that HIF-1α and VEGF expression in spinal cord injury areas after SCI is associated with the process of angiogenesis and improvement of local microcirculation. Erythropoietin is essential for recovering cognitive and memory disorders (following ischemic hypoxia) and appears to promote functional recovery after spinal cord injury. Also, after SCI, GM-CSF ensures the survival of dopaminergic neurons inhibits the formation of glial scars, having a neuroprotective role, too. The short intermittent experimental exposure to hypoxia produced respiratory, psychological, and motor function benefits in patients with incomplete deficits after SCI. In addition, deferoxamine treatment of rats with SCI induced a significant improvement in motor deficit, spared nerve tissue area, and electrophysiological conduction.

All these experimental findings justify the need to study the influence of hypoxia in the recovery of CNS disorders.

## Data Availability

Not applicable.

## Supplementary Materials

The following are available online at https://www.mdpi.com/article/10.3390/biomedicines10020481/s1, Table S1. Works selected through a customized (Gelu Onose, 2018) PEDro inspired filtering indirect quality classification.
