# Supplementary material for: Considerations about Hypoxic Changes in Neuraxis Tissue Injuries and Recovery
Source: Biomedicines. 2022 Feb 18;10(2):481. doi: 10.3390/biomedicines10020481 (PMC8962344; doi:10.3390/biomedicines10020481)
Supplement: Supplementary file 1 [file biomedicines-10-00481-s001.zip › biomedicines-1502852-supplementary.pdf]

biomedicines

MDPI

Systematic Review

Cellular and Molecular Targets for Non-invasive, Non-pharmacological therapeutic/rehabilitative interventions in Acute Ischemic Stroke

Table S1. Works selected through a customized (Gelu Onose, 2018) PEDro inspired filtering indirect quality classification.

No	*Article*	*Publication Year*	*Citation Count*	*Ref. no*	*PEDro score*
1	Claire Thornton, Bryan Leaw, Carina Mallard, Syam Nair, Masako Jinnai, Henrik Hagberg - Cell Death in the Developing Brain after Hypoxia-Ischemia - Front Cell Neurosci. 2017; 11: 248. Published online 2017 Aug 23. doi: 10.3389/fncel.2017.00248 DOI: 10.3389/fncel.2017.00248	2017	34	(11)	10
2	Nowak-Sliwinskaet al - Consensus guidelines for the use and interpretation of angiogenesis assays - Angiogenesis. Author manuscript; available in PMC 2019 Aug 1. Published in final edited form as: Angiogenesis. 2018 Aug; 21(3): 425–532. doi: 10.1007/s10456-018-9613-x DOI: 10.1007/s10456-018-9613-x.	2018	119	(23)	10
3	Servio H. Ramirez, Allison M. Andrews, Debayon Paul, Joel S. Pachter - Extracellular vesicles: mediators and biomarkers of pathology along CNS barriers - Fluids Barriers CNS. 2018; 15: 19. Published online 2018 Jul 1. doi: 10.1186/s12987-018-0104-7 DOI: 10.1186/s12987-018-0104-7	2018	35	(96)	10
4	Andrea Becerra-Calixto, Gloria P. Cardona-Gómez - The Role of Astrocytes in Neuroprotection after Brain Stroke: Potential in Cell Therapy - Front Mol Neurosci. 2017; 10: 88. Published online 2017 Apr 3. doi: 10.3389/fnmol.2017.00088 DOI: 10.3389/fnmol.2017.00088	2017	58	(61)	10
5	Markus Islinger, Alfred Voelkl, H. Dariush Fahimi, Michael Schrader - The peroxisome: an update on mysteries 2.0 - Histochem Cell Biol. 2018; 150(5): 443–471. Published online 2018 Sep 15. doi: 10.1007/s00418-018-1722-5 DOI: 10.1007/s00418-018-1722-5	2018	42	(62)	10
6	Gaforio et al. - Virgin Olive Oil and Health: Summary of the III International Conference on Virgin Olive Oil and Health Consensus Report, JAEN (Spain) 2018 - Nutrients. 2019 Sep; 11(9): 2039. Published online 2019 Sep 1. doi: 10.3390/nu11092039 DOI: 10.3390/nu11092039	2019	20	(132)	10
7	Cristina Angeloni, Marco Malaguti, Maria Cristina Barbalace, Silvana Hrelia - Bioactivity of Olive Oil Phenols in Neuroprotection - Int J Mol Sci. 2017 Nov; 18(11): 2230. Published online 2017 Oct 25. doi: 10.3390/ijms18112230 DOI: 10.3390/ijms18112230	2017	47	(133)	10
8	Federica Ciregia, Andrea Urbani, Giuseppe Palmisano - Extracellular Vesicles in Brain Tumors and Neurodegenerative Diseases - Front Mol Neurosci. 2017; 10: 276. Published online 2017 Aug 31. doi: 10.3389/fnmol.2017.00276 DOI: 10.3389/fnmol.2017.00276	2017	37	(98)	10
9	Manuela Pennisi, Giuseppe Lanza, Luca Falzone, Francesco Fisicaro, Raffaele Ferri, Rita Bella - SARS-CoV-2 and the Nervous System: From Clinical Features to Molecular Mechanisms - Int J Mol Sci. 2020 Aug; 21(15): 5475. Published online 2020 Jul 31. doi: 10.3390/ijms21155475 DOI: 10.3390/ijms21155475	2020	27	(89)	10
10	Yuan Yang, Lian Liu, Ishan Naik, Zachary Braunstein, Jixin Zhong, Boxu Ren - Transcription Factor C/EBP Homologous Protein in Health and Diseases - Front Immunol. 2017; 8: 1612. Published online 2017 Nov 27. doi: 10.3389/fimmu.2017.01612 DOI: 10.3389/fimmu.2017.01612	2017	40	(48)	10
11	Laurensia Yuniati, Blanca Scheijen, Laurens T. van der Meer, Frank N. van Leeuwen - Tumor suppressors BTG1 and BTG2: Beyond growth control - J Cell Physiol. 2019 May; 234(5): 5379–5389. Published online 2018 Oct 23. doi: 10.1002/jcp.27407 DOI: 10.1002/jcp.27407	2019	28	(44)	10
12	Martha Gschwandtner, Rupert Derler, Kim S. Midwood - More Than Just Attractive: How CCL2 Influences Myeloid Cell Behavior Beyond Chemotaxis - Front Immunol. 2019; 10: 2759. Published online 2019 Dec 13. doi: 10.3389/fimmu.2019.02759 DOI: 10.3389/fimmu.2019.02759	2019	38	(45)	10
13	Nunzio Iraci, Tommaso Leonardi, Florian Gessler, Beatriz Vega, Stefano Pluchino - Focus on Extracellular Vesicles: Physiological Role and Signalling Properties of Extracellular Membrane Vesicles - Int J Mol Sci. 2016 Feb; 17(2): 171. Published online 2016 Feb 6. doi: 10.3390/ijms17020171 DOI: 10.3390/ijms17020171	2016	74	(97)	10
14	Greenhalgh et al - Peripherally derived macrophages modulate microglial function to reduce inflammation after CNS injury - PLoS Biol. 2018 Oct; 16(10): e2005264. Published online 2018 Oct 17. doi: 10.1371/journal.pbio.2005264 DOI: 10.1371/journal.pbio.2005264	2018	48	(57)	10
15	Cai et al - Panoptic imaging of transparent mice reveals whole-body neuronal projections and skull-meninges connections - Nat Neurosci. Author manuscript; available in PMC 2019 Jun 30.Published in final edited form as: Nat Neurosci. 2019 Feb; 22(2): 317–327. Published online 2018 Dec 31. doi: 10.1038/s41593-018-0301-3 DOI: 10.1038/s41593-018-0301-3	2019	54	(14)	10
16	Magdalena Miranda, Juan Facundo Morici, María Belén Zanoni, Pedro Bekinschtein - Brain-Derived Neurotrophic Factor: A Key Molecule for Memory in the Healthy and the Pathological Brain - Front Cell Neurosci. 2019; 13: 363. Published online 2019 Aug 7. doi: 10.3389/fncel.2019.00363 DOI: 10.3389/fncel.2019.00363	2019	42	(113)	10

17	Rashida Ginwala, Raina Bhavsar, De Gaulle I. Chigbu, Pooja Jain, Zafar K. Khan - Potential Role of Flavonoids in Treating Chronic Inflammatory Diseases with a Special Focus on the Anti-Inflammatory Activity of Apigenin - Antioxidants (Basel) 2019 Feb; 8(2): 35. Published online 2019 Feb 5. doi: 10.3390/antiox8020035 DOI: 10.3390/antiox8020035	2019	24	(59)	10
18	Cheryl M. J. Tan, Peregrine Green, Nidi Tapoulal, Adam J. Lewandowski, Paul Leeson, Neil Herring - The Role of Neuropeptide Y in Cardiovascular Health and Disease - Front Physiol. 2018; 9: 1281. Published online 2018 Sep 19. doi: 10.3389/fphys.2018.01281 DOI: 10.3389/fphys.2018.01281	2018	23	(126)	9
19	Emine Şekerdağ, Ihsan Solaroğlu, Yasemin Gürsoy-Özdemir - Cell Death Mechanisms in Stroke and Novel Molecular and Cellular Treatment Options - Curr Neuropharmacol. 2018 Nov; 16(9): 1396–1415. Published online 2018 Nov. doi: 10.2174/1570159X16666180302115544 DOI: 10.2174/1570159X16666180302115544	2018	23	(93)	9
20	Justin Hou Ming Yung, Adria Giacca - Role of c-Jun N-terminal Kinase (JNK) in Obesity and Type 2 Diabetes - Cells. 2020 Mar; 9(3): 706. Published online 2020 Mar 13. doi: 10.3390/cells9030706 DOI: 10.3390/cells9030706	2020	12	(128)	9
21	Dong Seok Kim, Ho-Il Choi, Yun Wang, Yu Luo, Barry J. Hoffer, Nigel H. Greig - A New Treatment Strategy for Parkinson's Disease through the Gut–Brain Axis: The Glucagon-Like Peptide-1 Receptor Pathway - Cell Transplant. 2017 Sep; 26(9): 1560–1571. Published online 2017 Nov 8. doi: 10.1177/0963689717721234 DOI: 10.1177/0963689717721234	2017	29	(66)	9
22	Łukasz A. Poniatowski, Piotr Wojdasiewicz, Maciej Krawczyk, Dariusz Szukiewicz, Robert Gasik, Łukasz Kubaszewski, Iwona Kurkowska-Jastrzębska - Analysis of the Role of CX3CL1 (Fractalkine) and Its Receptor CX3CR1 in Traumatic Brain and Spinal Cord Injury: Insight into Recent Advances in Actions of Neurochemokine Agents - Mol Neurobiol. 2017; 54(3): 2167–2188. Published online 2016 Mar 1. doi: 10.1007/s12035-016-9787-4 DOI: 10.1007/s12035-016-9787-4	2017	25	(100)	8
23	Jae Young Kim, Jong Youl Kim, Jae Hwan Kim, Hosung Jung, Won Taek Lee, Jong Eun Lee - Restorative Mechanism of Neural Progenitor Cells Overexpressing Arginine Decarboxylase Genes Following Ischemic Injury - Exp Neurobiol. 2019 Feb; 28(1): 85–103. Published online 2019 Jan 21. doi: 10.5607/en.2019.28.1.85 DOI: 10.5607/en.2019.28.1.85	2019	16	(104)	8
24	Zong-Jian Liu, Yuan-Yuan Ran, Shu-Yan Qie, Wei-Jun Gong, Fu-Hai Gao, Zi-Tong Ding, Jia-Ning Xi - Melatonin protects against ischemic stroke by modulating microglia/macrophage polarization toward anti-inflammatory phenotype through STAT3 pathway - CNS Neurosci Ther. 2019 Dec; 25(12): 1353–1362. Published online 2019 Dec 2. doi: 10.1111/cns.13261 DOI: 10.1111/cns.13261	2019	16	(60)	8
25	James P. Barrett, Rebecca J. Henry, Sonia Villapol, Bogdan A. Stoica, Alok Kumar, Mark P. Burns, Alan I. Faden, David J. Loane - NOX2 deficiency alters macrophage phenotype through an IL-10/STAT3 dependent mechanism: implications for traumatic brain injury - J Neuroinflammation. 2017; 14: 65. Published online 2017 Mar 24. doi: 10.1186/s12974-017-0843-4 DOI: 10.1186/s12974-017-0843-4	2017	26	(58)	8
26	Samuel W. Cramer, Clark C. Chen - Photodynamic Therapy for the Treatment of Glioblastoma - Front Surg. 2019; 6: 81. Published online 2020 Jan 21. doi: 10.3389/fsurg.2019.00081 DOI: 10.3389/fsurg.2019.00081	2020	10	(140)	8
27	Andrew D. Miller, James F. Zachary - Nervous System - Pathologic Basis of Veterinary Disease. 2017 : 805–907.e1. Published online 2017 Feb 17. doi: 10.1016/B978-0-323-35775-3.00014-X DOI: 10.1016/B978-0-323-35775-3.00014-X	2017	22	(56)	7
28	Francisco J. Carvajal, Hayley A. Mattison, Waldo Cerpa - Role of NMDA Receptor-Mediated Glutamatergic Signaling in Chronic and Acute Neuropathologies - Neural Plast. 2016; 2016: 2701526. Published online 2016 Aug 18. doi: 10.1155/2016/2701526 DOI: 10.1155/2016/2701526	2016	27	(87)	7
29	Carolina M. Gorgulho, Graziela G. Romagnoli, Rosh Bharthi, Michael T. Lotze - Johnny on the Spot-Chronic Inflammation Is Driven by HMGB1 - Front Immunol. 2019; 10: 1561. Published online 2019 Jul 11. doi: 10.3389/fimmu.2019.01561 DOI: 10.3389/fimmu.2019.01561	2019	13	(65)	7
30	Masaru Tanaka, József Toldi, László Vécsei - Exploring the Etiological Links behind Neurodegenerative Diseases: Inflammatory Cytokines and Bioactive Kynurenines - Int J Mol Sci. 2020 Apr; 21(7): 2431. Published online 2020 Mar 31. doi: 10.3390/ijms21072431 DOI: 10.3390/ijms21072431	2020	9	(92)	7
31	Alexander A. Ilyasov, Carolanne E. Milligan, Emily P. Pharr, Allyn C. Howlett - The Endocannabinoid System and Oligodendrocytes in Health and Disease - Front Neurosci. 2018; 12: 733. Published online 2018 Oct 26. doi: 10.3389/fnins.2018.00733 DOI: 10.3389/fnins.2018.00733	2018	15	(136)	6
32	Wensheng Lin, Sarrabeth Stone - Unfolded protein response in myelin disorders - Neural Regen Res. 2020 Apr; 15(4): 636–645. Published online 2019 Oct 18. doi: 10.4103/1673-5374.266903 DOI: 10.4103/1673-5374.266903	2020	8	(101)	6
33	Li Zhou, Ping Chen, Yating Peng, Ruoyun Ouyang - Role of Oxidative Stress in the Neurocognitive Dysfunction of Obstructive Sleep Apnea Syndrome - Oxid Med Cell Longev. 2016; 2016: 9626831. Published online 2016 Sep 28. doi: 10.1155/2016/9626831 DOI: 10.1155/2016/9626831	2016	22	(119)	6

34	Fahimeh Shahabipour, Nastaran Barati, Thomas P. Johnston, Giuseppe Derosa, Pamela Maffioli, Amirhossein Sahebkar - Exosomes: Nanoparticulate tools for RNA interference and drug delivery - J Cell Physiol. 2017 Jul; 232(7): 1660–1668. Published online 2017 Jan 31. doi: 10.1002/jcp.25766 DOI: 10.1002/jcp.25766	2017	19	(90)	6
35	Ralf Weiskirchen - Hepatoprotective and Anti-fibrotic Agents: It's Time to Take the Next Step - Front Pharmacol. 2015; 6: 303. Published online 2016 Jan 7. doi: 10.3389/fphar.2015.00303 DOI: 10.3389/fphar.2015.00303	2016	22	(79)	6
36	Po-Yuan Ke - Diverse Functions of Autophagy in Liver Physiology and Liver Diseases - Int J Mol Sci. 2019 Jan; 20(2): 300. Published online 2019 Jan 13. doi: 10.3390/ijms20020300 DOI: 0.3390/ijms20020300	2019	9	(121)	5
37	John G. Geisler - 2,4 Dinitrophenol as Medicine - Cells. 2019 Mar; 8(3): 280. Published online 2019 Mar 23. doi: 10.3390/cells8030280 DOI: 10.3390/cells8030280	2019	10	(77)	5
38	Morya et al - Beyond the target area: an integrative view of tDCS-induced motor cortex modulation in patients and athletes - J Neuroeng Rehabil. 2019; 16: 141. Published online 2019 Nov 15. doi: 10.1186/s12984-019-0581-1 DOI: 10.1186/s12984-019-0581-1	2019	9	(94)	5
39	Omar de Faria, Jr., David G. Gonsalvez, Madeline Nicholson, Junhua Xiao - Activity-dependent central nervous system myelination throughout life - J Neurochem. 2019 Feb; 148(4): 447–461. Published online 2018 Nov 12. doi: 10.1111/jnc.14592 DOI: 10.1111/jnc.14592	2019	8	(82)	4
40	Andrew R. Clark, Michael Ohlmeyer - Protein phosphatase 2A as a therapeutic target in inflammation and neurodegeneration - Pharmacol Ther. 2019 Sep; 201: 181–201. doi: 10.1016/j.pharmthera.2019.05.016 DOI: 10.1016/j.pharmthera.2019.05.016	2019	7	(55)	4
41	Hyemin Gu, Sang Mi Han, Kwan-Kyu Park - Therapeutic Effects of Apamin as a Bee Venom Component for Non-Neoplastic Disease - Toxins (Basel) 2020 Mar; 12(3): 195. Published online 2020 Mar 19. doi: 10.3390/toxins12030195 DOI: 10.3390/toxins12030195	2020	5	(139)	4
42	Rosaliana Libro, Sabrina Giacoppo, Thangavelu Soundara Rajan, Placido Bramanti, Emanuela Mazzon - Natural Phytochemicals in the Treatment and Prevention of Dementia: An Overview - Molecules. 2016 Apr; 21(4): 518. Published online 2016 Apr 21. doi: 10.3390/molecules21040518 DOI: 10.3390/molecules21040518	2016	15	(134)	4
43	Raluca Ioana Teleanu, Cristina Chircov, Alexandru Mihai Grumezescu, Adrian Volceanov, Daniel Mihai Teleanu - Antioxidant Therapies for Neuroprotection—A Review - J Clin Med. 2019 Oct; 8(10): 1659. Published online 2019 Oct 11. doi: 10.3390/jcm8101659 DOI: 10.3390/jcm8101659	2019	7	(135)	4
44	Landon J. Rohowetz, Jacob G. Kraus, Peter Koulen - Reactive Oxygen Species-Mediated Damage of Retinal Neurons: Drug Development Targets for Therapies of Chronic Neurodegeneration of the Retina - Int J Mol Sci. 2018 Nov; 19(11): 3362. Published online 2018 Oct 27. doi: 10.3390/ijms19113362 DOI: 10.3390/ijms19113362	2018	11	(78)	4
45	Unai Galicia-Garcia, Asier Benito-Vicente, Shifa Jebari, Asier Larrea-Sebal, Haziq Siddiqi, Kepa B. Uribe, Helena Ostolaza, César Martín - Pathophysiology of Type 2 Diabetes Mellitus - Int J Mol Sci. 2020 Sep; 21(17): 6275. Published online 2020 Aug 30. doi: 10.3390/ijms21176275 DOI: 10.3390/ijms21176275	2020	5	(88)	4
